# Supplementary material for: Golgi pH homeostasis stabilizes the lysosomal membrane through N-glycosylation of membrane proteins
Source: Life Sci Alliance. 2024 Jul 30;7(10):e202402677. doi: 10.26508/lsa.202402677 (PMC11289521; doi:10.26508/lsa.202402677)

Full blot images for Figure 1A

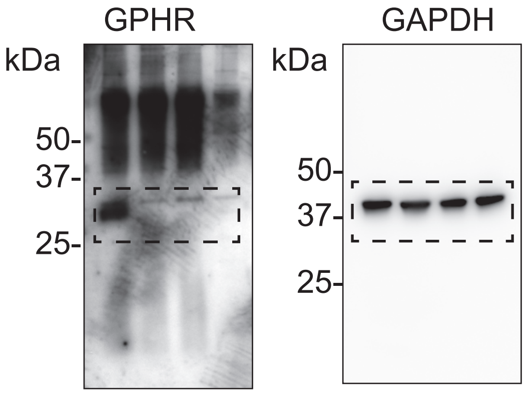

Full blot images for Figure 1D

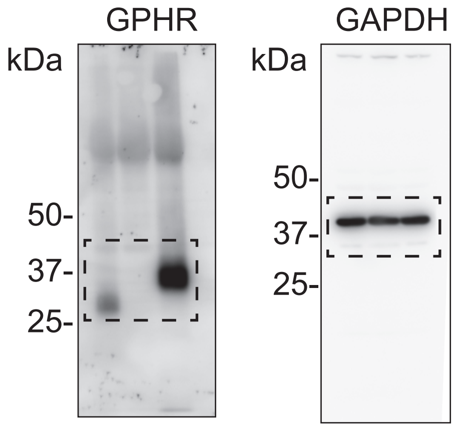

Full blot images for Figure 2C

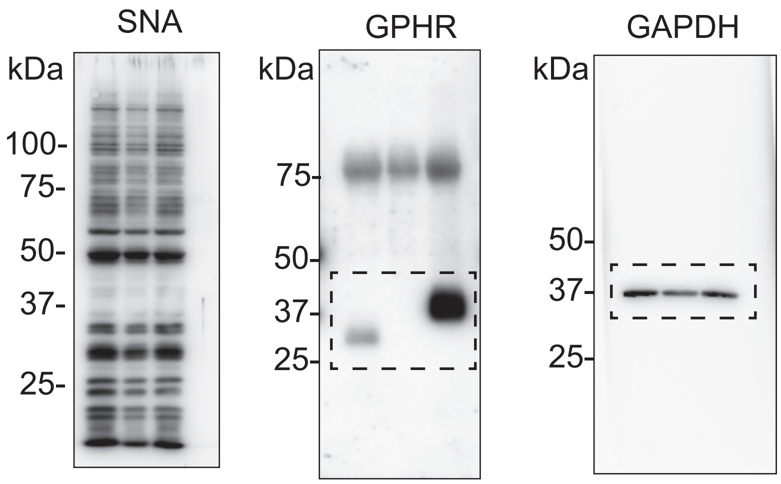

Full blot images for Figure 2E

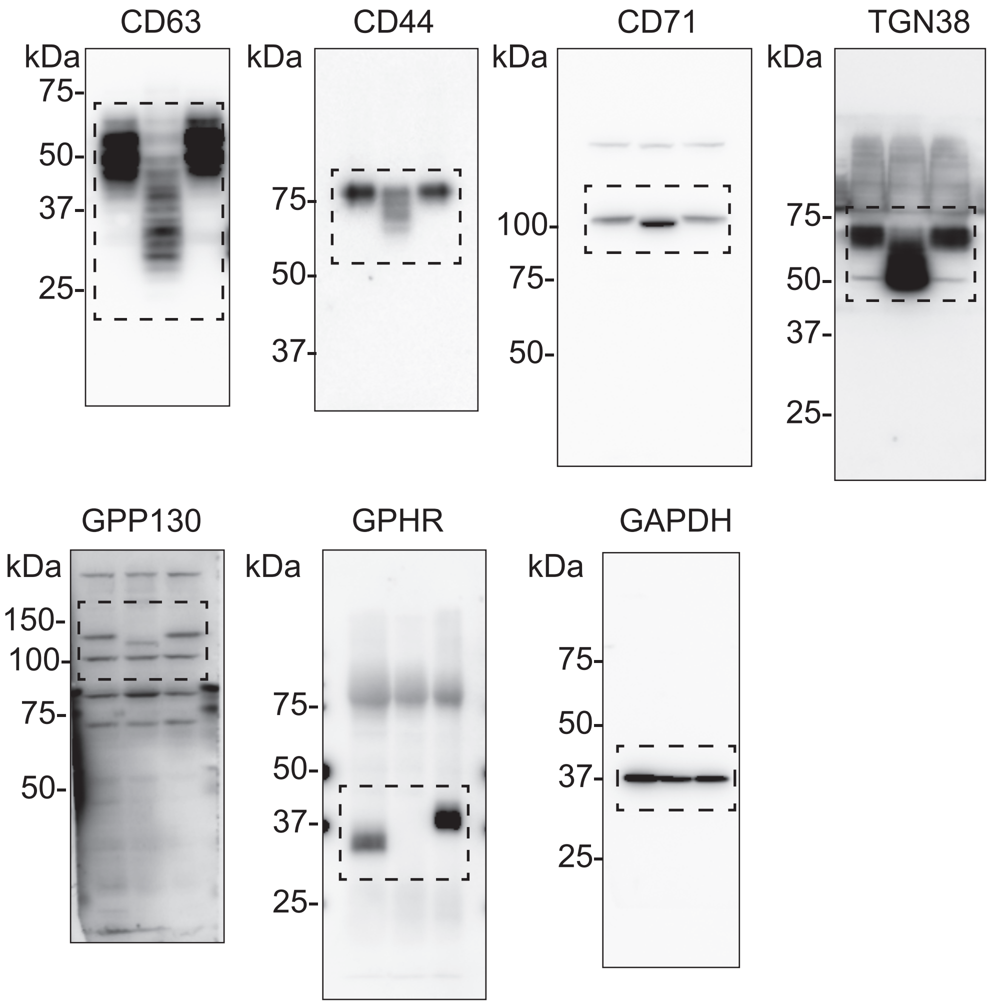

Supplement: Supplementary file 1 [file LSA-2024-02677_SdataF1_F2.pdf]
