# Supplementary material for: Golgi pH homeostasis stabilizes the lysosomal membrane through N-glycosylation of membrane proteins
Source: Life Sci Alliance. 2024 Jul 30;7(10):e202402677. doi: 10.26508/lsa.202402677 (PMC11289521; doi:10.26508/lsa.202402677)

Full blot images for Supplemental Figure 1A

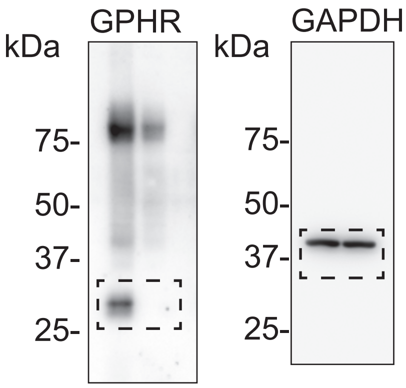

Full blot images for Supplemental Figure 2A

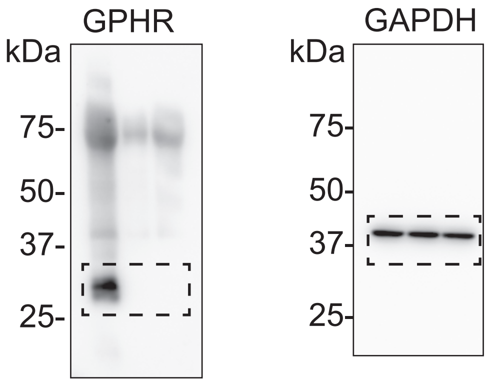

Full blot images for Supplemental Figure 3

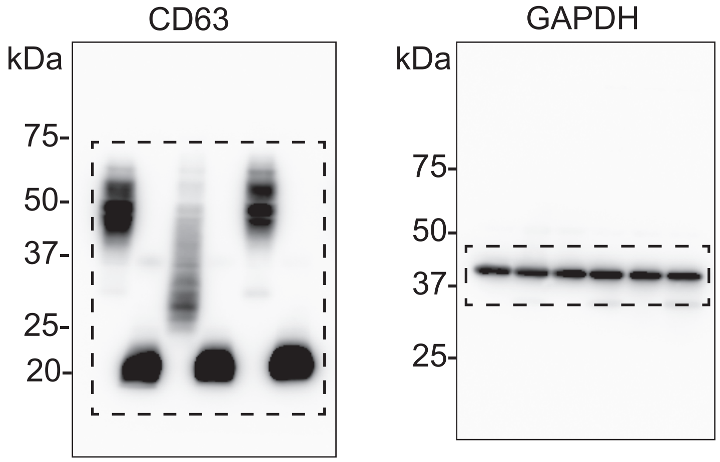

Full blot images for Supplemental Figure 4A

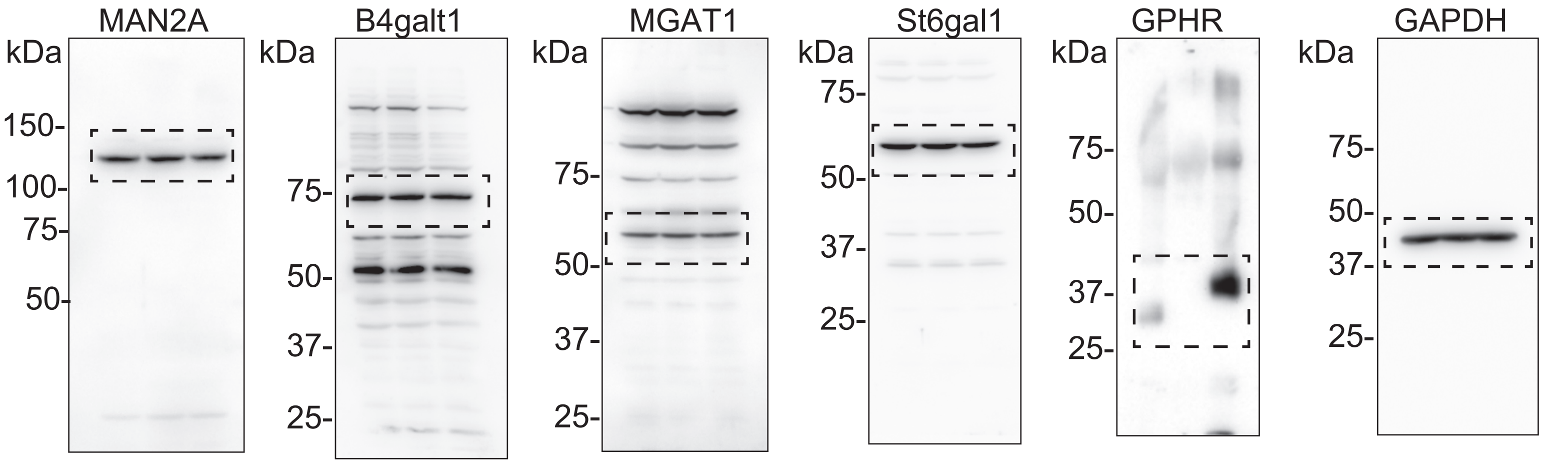

Supplement: Supplementary file 2 [file LSA-2024-02677_SdataFS1_FS2_FS3_FS4.pdf]
