# Supplementary material for: Golgi pH homeostasis stabilizes the lysosomal membrane through N-glycosylation of membrane proteins
Source: Life Sci Alliance. 2024 Jul 30;7(10):e202402677. doi: 10.26508/lsa.202402677 (PMC11289521; doi:10.26508/lsa.202402677)

Full blot images for Supplemental Figure 5A

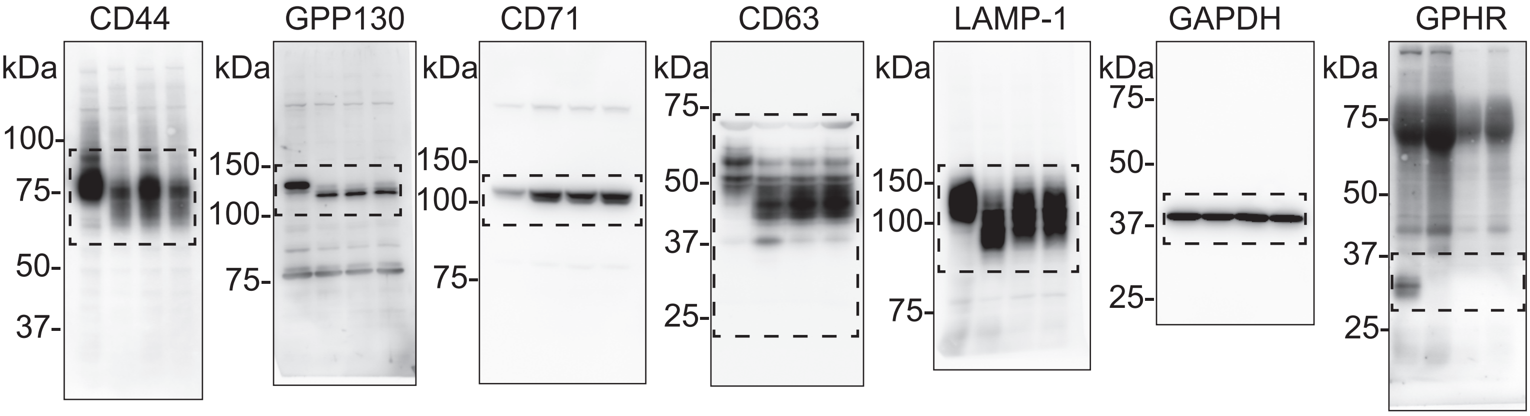

Full blot images for Supplemental Figure 5B

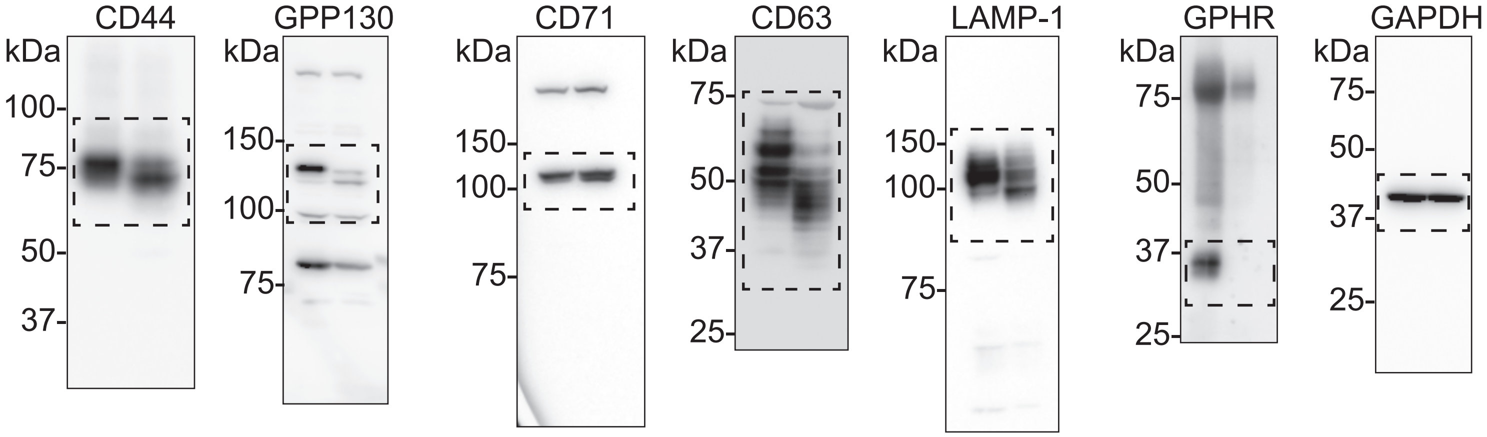

Full blot images for Supplemental Figure 5C

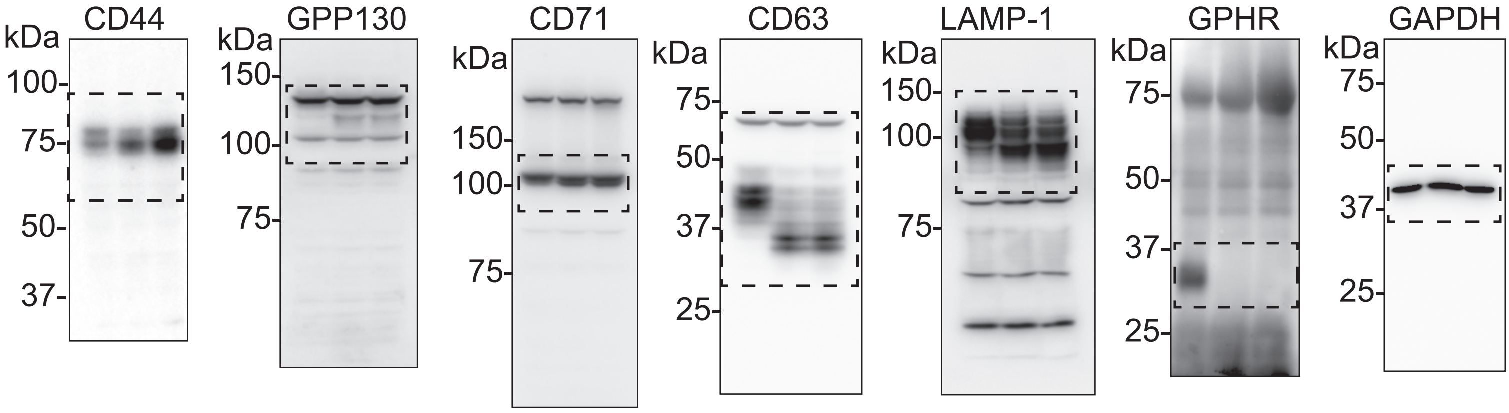

Supplement: Supplementary file 3 [file LSA-2024-02677_SdataFS5.pdf]
