# Supplementary figures and images for: Golgi pH homeostasis stabilizes the lysosomal membrane through N-glycosylation of membrane proteins
Source: Life Sci Alliance. 2024 Jul 30;7(10):e202402677. doi: 10.26508/lsa.202402677 (PMC11289521; doi:10.26508/lsa.202402677)

Full blot images for Figure 5A

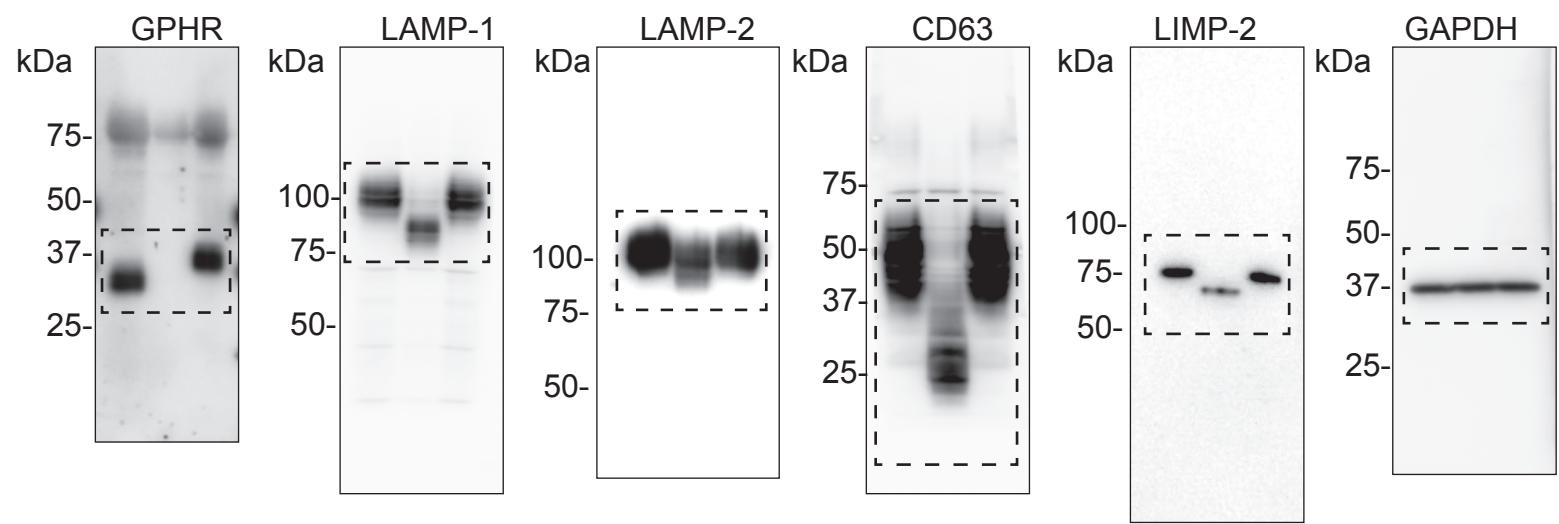

Full blot images for Figure 5B

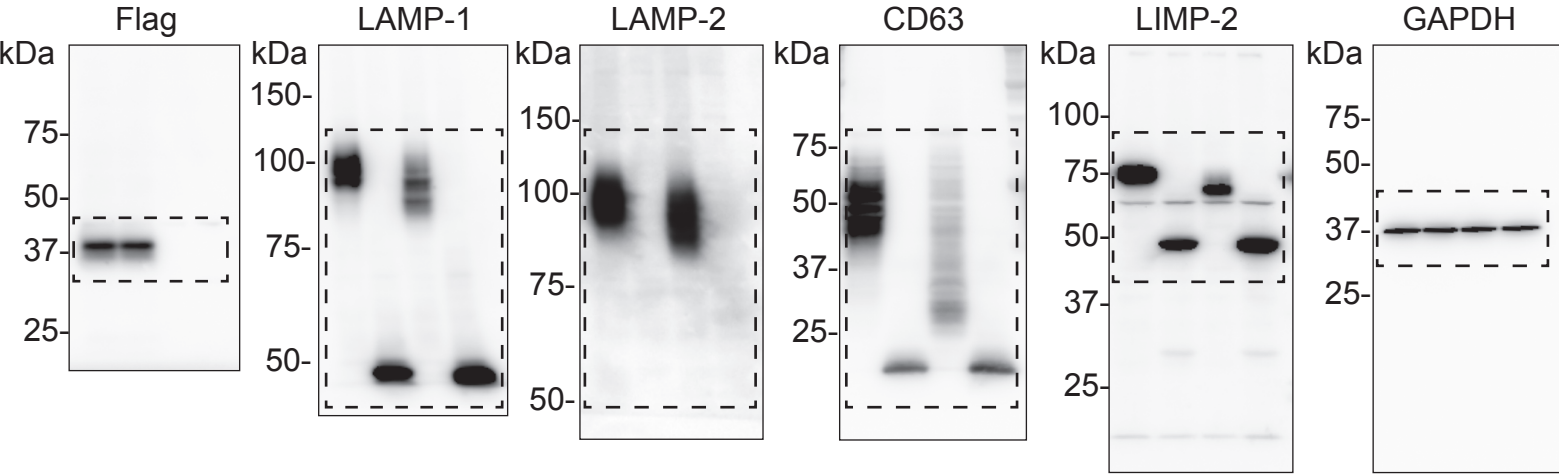

Full blot images for Figure 5C

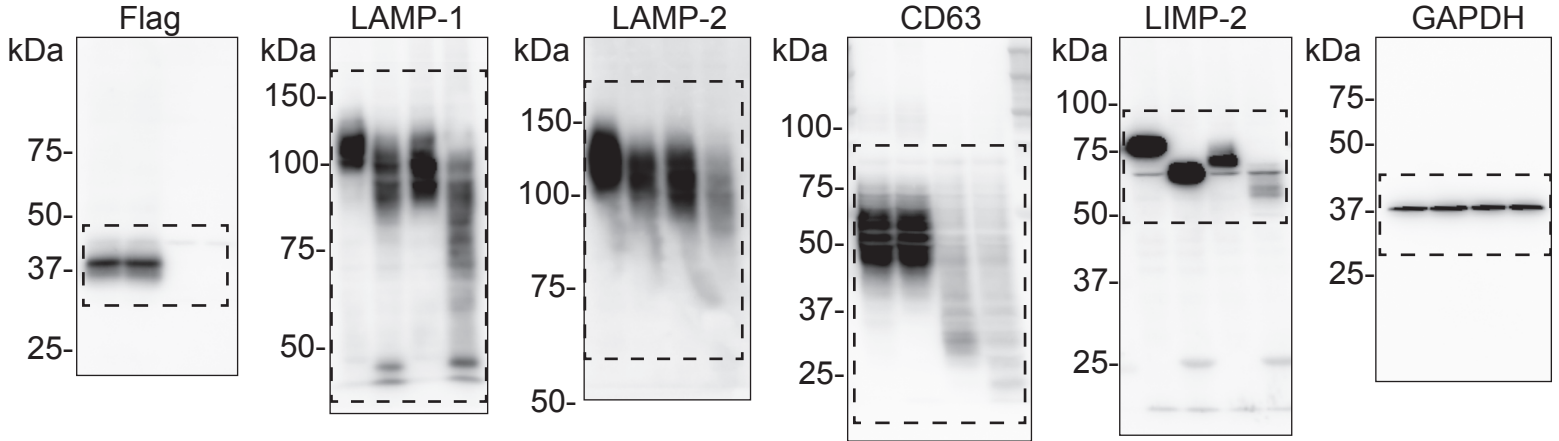

Supplement: Supplementary file 8 [file LSA-2024-02677_SdataF5.pdf]

Full blot images for Figure 8C

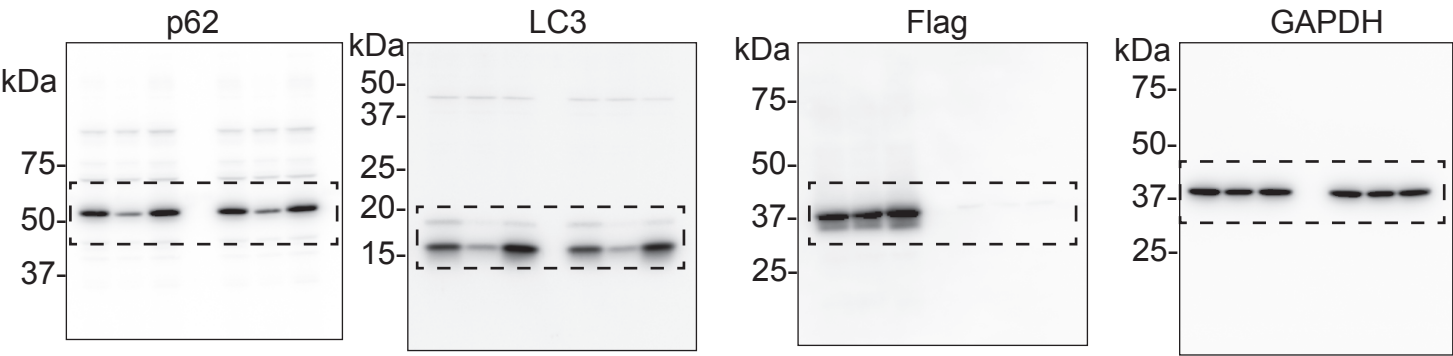

Full blot images for Figure 9A

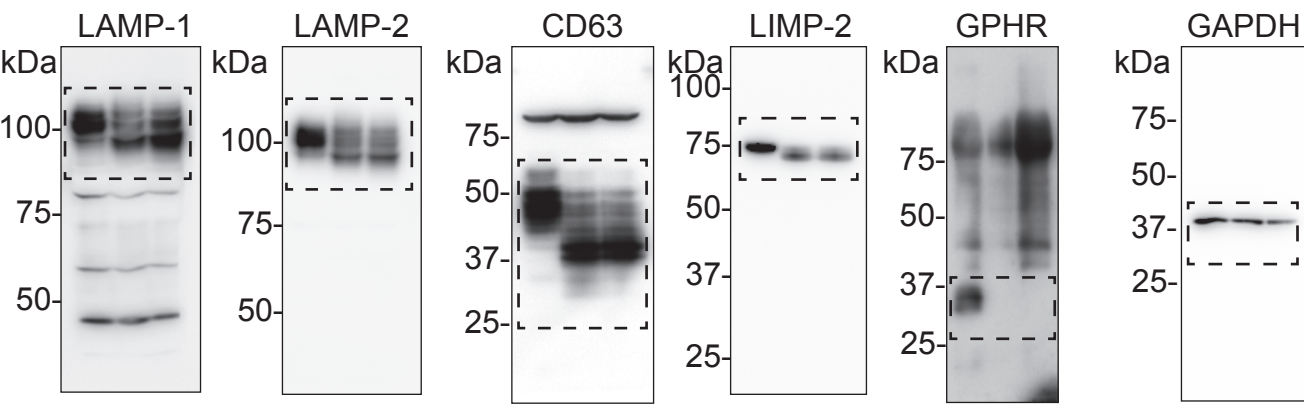

Full blot images for Figure 10A

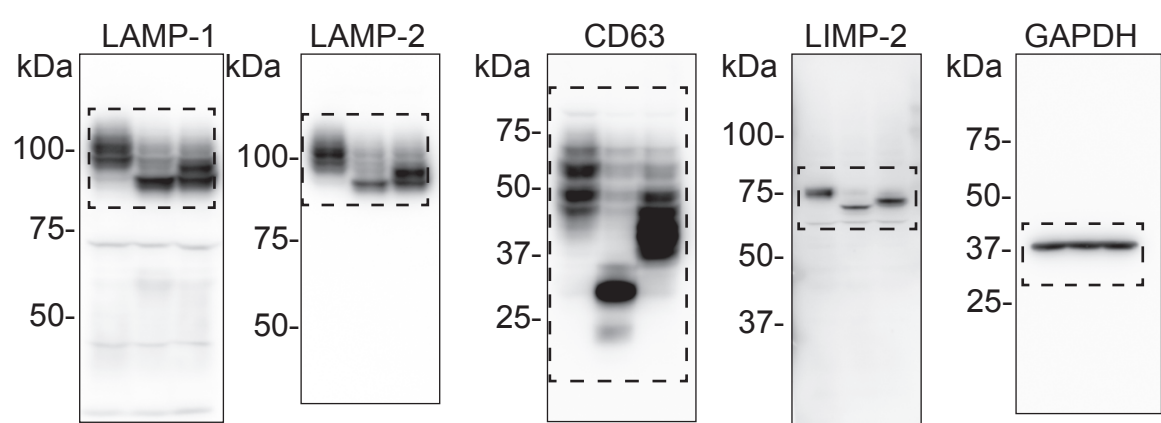

Supplement: Supplementary file 9 [file LSA-2024-02677_SdataFS8_FS9_FS10.pdf]
